# Efficacy and safety of whole-body vibration therapy for post-stroke spasticity: A systematic review and meta-analysis

**DOI:** 10.3389/fneur.2023.1074922

**Published:** 2023-01-26

**Authors:** Qi Zhang, Shuqi Zheng, Shuiyan Li, Yuting Zeng, Ling Chen, Gege Li, Shilin Li, Longlong He, Shuping Chen, Xiaoyan Zheng, Jihua Zou, Qing Zeng

**Affiliations:** ^1^School of Rehabilitation Sciences, Southern Medical University, Guangzhou, China; ^2^Department of Rehabilitation Medicine, Zhujiang Hospital, Southern Medical University, Guangzhou, China; ^3^Faculty of Health and Social Sciences, The Hong Kong Polytechnic University, Hong Kong, China

**Keywords:** whole body vibration therapy, stroke, muscle spasticity, rehabilitation, meta-analysis

## Abstract

**Background:**

One of the main objectives of stroke rehabilitation is to alleviate post-stroke spasticity. Over the recent years, many studies have explored the potential benefits of whole-body vibration (WBV) treatment for post-stroke spasticity, but it is still controversial.

**Objective:**

The current study aims to assess the efficacy and safety of WBV for post-stroke spasticity and determine the appropriate application situation.

**Methods:**

From their establishment until August 2022, the following databases were searched: PubMed, Web of Science, Embase, Cochrane Central Register of Controlled Trials (CENTRAL), Medline, China National Knowledge Infrastructure (CNKI), and Wanfang. Only randomized controlled trials (RCTs) that were published in either English or Chinese were taken into consideration. We independently filtered the research, gathered the data from the studies, and evaluated the research quality (Cochrane RoB tool) and the overall evidence quality (GRADE). Rev Man 5.4 software was utilized to conduct statistical analysis.

**Results:**

In this analysis, 11 RCTs with 475 patients that reported on the effectiveness of WBV therapy for post-stroke spasticity were taken into account. Compared to the control groups, the results revealed that WBV combined with conventional rehabilitation at a vibration frequency lower than 20 Hz (SMD = −0.58, 95% CI: −0.98 to −0.19, *P* = 0.004) was more effective in relieving upper (SMD = −0.53, 95% CI: −1.04 to 0.03, *P* = 0.03) and lower limb spasticity (SMD = −0.21, 95% CI: −0.40 to −0.01, *P* = 0.04); similarly, it was superior for patients aged under 60 years (SMD = −0.41, 95% CI: −0.66 to −0.17, *P* = 0.0008) with acute and subacute stroke (SMD = −0.39, 95% CI: −0.68 to −0.09, *P* = 0.01). The valid vibration for reducing spasticity was found to last for 10 min (SMD = −0.41, 95% CI: −0.75 to −0.07, *P* = 0.02). None of the included studies revealed any serious adverse impact.

**Conclusion:**

Moderate-quality evidence demonstrated when WBV was used as an adjuvant, vibration <20 Hz for 10 min was effective and secure in treating upper and lower limb spasticity in patients with acute and subacute stroke under the age of 60 years.

**Systematic review registration:**

https://www.crd.york.ac.uk/PROSPERO/, identifier: CRD42022293951.

## 1. Introduction

Throughout the world, stroke is indeed the second leading cause of death and the third leading cause of disability (1). According to statistics, about 5.5 million people die from stroke every year, and the risk of stroke in adults is as high as 24.9% (2). As of 2019, China ranked first in stroke incidence globally, with ~17 million people over 40 years of age affected by stroke (2). Spasticity is the most frequent after-stroke consequence, affecting about 40% of patients within days or weeks, and resulting in adverse effects, such as pain, immobility, and muscular contracture, which severely reduce the quality of life (3). Direct finance due to post-stroke spasticity (such as hospitalization, drugs, and health professional services) is about four times more than those who do not have spasticity (4). Thus, spasticity management is an essential component of stroke rehabilitation.

Spasticity can be treated in various ways, such as by administering botulinum toxin injections (5), which is considered the most widely used local treatment of spasticity. Rehabilitation interventions such as stretching of the muscles (5), aquatic exercise (6, 7), mirror therapy (8, 9), ultrasound therapy (10), electrical stimulation (11), and extracorporeal shock wave therapy (12) have also been shown to be useful. Recently, whole-body vibration (WBV) was suggested as a potential therapeutic approach to address spasticity in stroke survivors (13, 14). WBV was recognized as a non-invasive, easily accepted and operated, and well-tolerated technique in which an individual stands on a vibratory board that emits sinusoidal oscillations through the feet to the entire body (15).

Several possible mechanisms have been proposed to explain decreased post-stroke spasticity during exposure to WBV. One proposed mechanism (16) hypothesized that WBV may suppress the synaptic transmission between Ia afferent and motor neurons by inducing presynaptic inhibition, then inhibiting the excitation of spastic muscles, thereby reducing muscle tension. Another way in which vibration has this effect was through the “busy hypothesis,” in which the Ia discharge becomes locked to vibration and was subsequently unable to accurately convey the stretch-induced volley due to the entrained action potentials and the high vibration frequency in the Ia fibers (17). Nevertheless, the mechanism of action on spasticity is complicated, and the straightforward monosynaptic reflex or a single route cannot fully account for the anti-spasticity effect of WBV. Many transcranial magnetic stimulation studies (18–20) have demonstrated that WBV also has an impact on the brain's central nervous system. By altering the brain's central nervous system or reducing spinal cord excitability, spasticity of the affected lower limb may alleviate (21). Miyara et al. (22) found that WBV could increase cortical excitability by functional near-infrared spectroscopy. Thus, WBV is thought to be a potential approach for treating spasticity in patients with stroke.

Despite some randomized controlled experiments claiming that WBV reduces post-stroke spasticity (23–25), some other researchers reported it was not observed a benefit of WBV in reducing muscular spasticity (26). Therefore, how WBV impacts post-stroke spasticity is still a debate. This study aims to compile randomized controlled studies that have already been published, analyze the efficacy and safety of WBV systematically, and offer more thorough and rigorous proof for the application of WBV to treat post-stroke spasticity.

## 2. Methods

This meta-analysis was performed in accordance with the PRISMA guidelines (27). The protocol has a PROSPERO registration (registration code CRD42022293951). Every study was based on previously published research; therefore, neither written consent nor ethical clearance was required.

### 2.1. Eligibility criteria

The following eligibility requirements have to be satisfied for an original study to be taken into account in our meta-analysis in accordance with the PICOS recommendation (28).

(1) Study types: English or Chinese-language RCTs. (2) Participants types: The population of interest included patients with a diagnosis of post-stroke spasticity and those who give consent to WBV treatment. (3) Interventions: control subjects received sham vibration or identical interventions in both groups, whereas those in the experimental class received WBV or WBV in addition to other therapy (where WBV was an add-on in one group) more than one session. (4) Outcomes: In all included trials, to gauge the degree of spasticity, the Modified Ashworth Scale (MAS) was applied. The outcome measures included those adverse effects that patients experienced during the follow-up time. No distinction was made between outcome data provided as a primary or secondary variable.

### 2.2. Search strategy

According to the PRISMA guidelines and PICOS design, two reviewers (QZh and SYL) independently carried out a thorough literature search of the PubMed (from 1996), Web of Science (from 1997), Embase (from 1980), Cochrane Central Register of Controlled Trials (CENTRAL) (Cochrane Library, latest issue), Medline (from 1948), China National Knowledge Infrastructure (CNKI) (from 1999), and Wanfang (from 2001) for published researches in English or Chinese on WBV for post-stroke spasticity from inception to August 2022. We combined free-text terms with regulated vocabulary (i.e., medical subject headings) as our search approach. The keywords used for searching included WBV, WBVT, vibration training, whole-body vibration, stroke, cerebrovascular accident, brain vascular accident, cerebrovascular strokes, muscle spasticity, spastic, muscle spasm, and muscular spasm. Only English and Chinese were the available languages. In order to satisfy each database's unique requirements, search strategies were changed. The PubMed search approach is shown in Supplementary Figure S1. In addition, we carefully looked over the retrieved publications' reference lists in an effort to find more pertinent studies.

### 2.3. Literature selection

Two reviewers, YZ and LC, carried out the search strategy and retrieved the abstracts of pertinent publications. The software Endnote X9 was then used to import all of the publications, and duplicate publications were eliminated. After examining the article titles, abstracts, and entire texts, we independently obtained the publications that matched the inclusion and exclusion criteria. Conflicts over which studies to include or exclude were settled through consensus discussions between YZ and LC or through consulting another reviewer (GL).

### 2.4. Data extraction

YZ and LC separately extracted data from the included randomized clinical trials. According to the recommendations for WBV intervention reporting (29), we extracted and cross-checked the vibration parameters (frequency, amplitude, and duration), protocol characteristics (intervention methods, spasticity sites, positions, follow-up time, etc.), adverse effects, initial author, the publication year, mean age, and course of a stroke. A third researcher (GL) was consulted for any inconsistent data.

### 2.5. Risk of bias assessment

Using the Cochrane Risk of Bias (RoB) methodology (30), two reviewers evaluated the methodological quality of all included publications. The following domains were evaluated: attrition prejudice (incomplete outcome data), detecting bias (blinding of outcome evaluation), selection bias (random sequence generation and allocation concealment), performance bias (blinding of participants and staff), and reporting bias (selective reporting) (31). Three levels of outcomes (low risk, high risk, and unclear) from the evaluation were established (30). In addition, the discrepancies were cleared out by intragroup conversations and by getting in touch with the authors to clarify specifics with the third-party arbitrator.

### 2.6. Level of evidence

To evaluate the overall evidence quality, we used the GRADE method (32). Study constraints, indirectness of evidence, unexplained heterogeneity or discrepancy of results, imprecision of outcomes, and a high chance of publication bias are five conditions that determine the quality of the evidence (33). The summary of data tables was available on the GRADEpro or GRADEpro GDT website (www.gradepro.org) (34). The quality of the evidence and the RoB were evaluated independently by two reviewers (QZh and SZ). When in question, the decision was made after consulting another reviewer (SYL).

### 2.7. Statistical analysis

The statistics analysis was completed using Review Manager 5.4 software. In order to combine trials that measured the same result using several scales, the analysis of continuous outcomes was done by computing the SMD with 95% CI. If there were many experimental or sham stimulation groups included in the study, we pooled the experimental or control groups in an attempt to eliminate the number of comparisons (35). A fixed-effects model was employed for no or small heterogeneity studies (i.e., *P* ≥ 0.1 or *I*^2^ ≤ 50%), whereas the random-effects model was used for high heterogeneity studies (i.e., *P* < 0.1 or *I*^2^ > 50%). The likelihood of publication bias was evaluated for a meta-analysis using a funnel plot analysis. In addition, we divided the data into subgroups based on age, location of the vibration, frequency, and duration. When the literature only provided the median or range of data, the mean and standard deviation were estimated according to a validated mathematical formula proposed by Luo et al. (36).

## 3. Results

### 3.1. Literature selection

**Figure 1:**
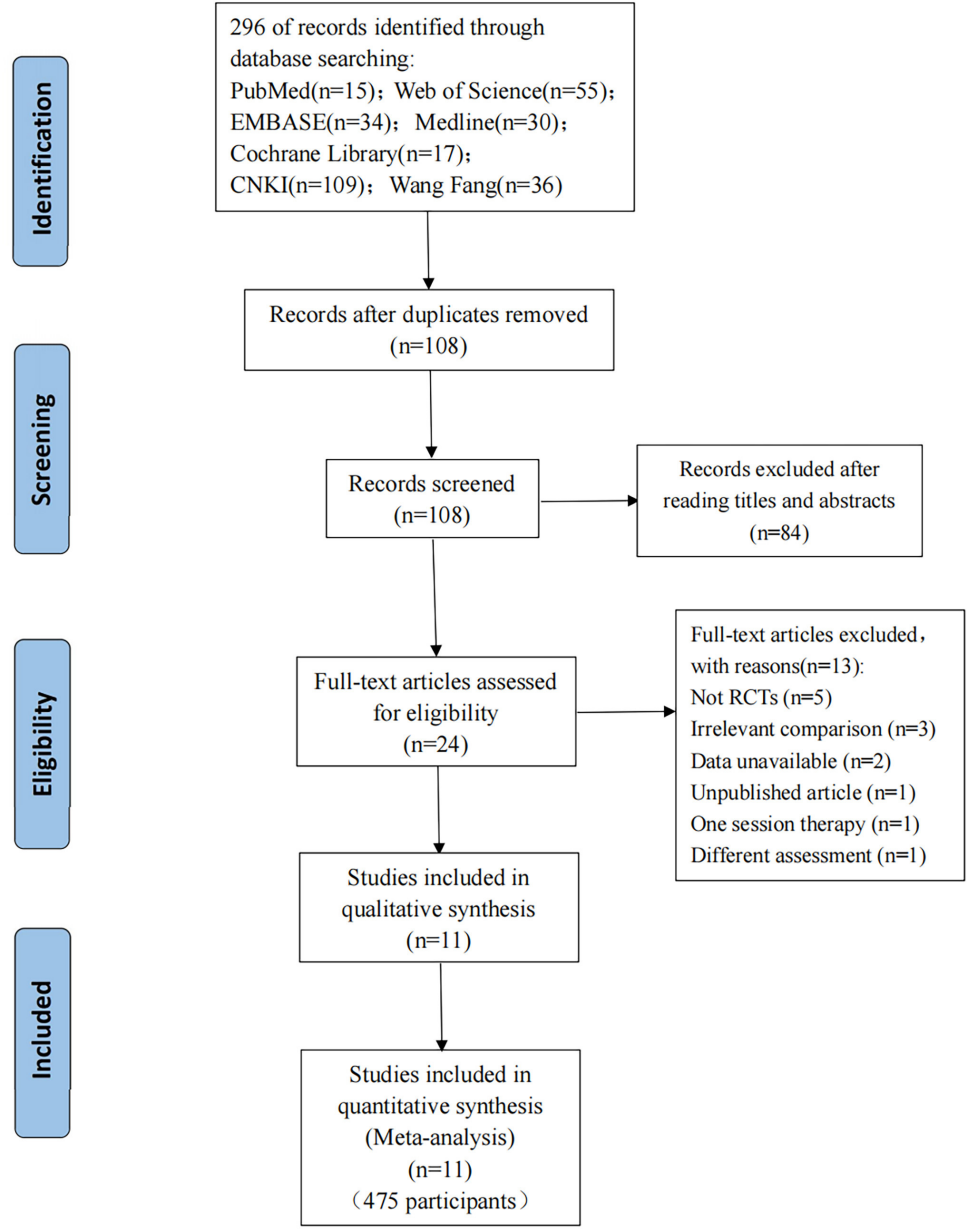
PRISMA flow diagram presenting the study selection process.

A total of 296 publications were identified (15 articles from PubMed, 55 articles from Web of Science, 34 articles from Embase, 30 articles from Medline, 17 articles from CENTRAL, 109 articles from CNKI, and 36 articles from Wang Fang); these were added to Endnote X9 (Clarivate Analytics). A total of 108 articles kept the removal of duplicates. A total of 24 publications were left after going over the titles and abstracts and eliminating reviews and other irrelevant studies. Finally, 13 of the 24 articles that were evaluated for eligibility were rejected: five because they were not RCTs; three because the aim of the comparison was to the combined efficacy of WBV plus additional treatments, not the effect of WBV alone; two because needed data was unavailable; one because it was an unpublished article; one because it had only one session and one because the outcome was not assessed by MAS. Thus, 11 articles (13, 23, 26, 37–44) were ultimately included in this meta-analysis. In Figure 1, the entire flowchart of the study screening process is displayed.

### 3.2. Characteristics of the included studies

Our research comprised a total of 11 studies covering 475 patients with post-stroke spasticity (252 participants in the experimental group, and 223 in the control group). In the experimental group, the intervention was WBV or conventional treatment supplemented with WBV, whereas the control group received sham vibration or conventional treatment. For spasticity sites, nine articles (13, 23, 26, 37, 38, 40–42, 44) included patients with lower limb spasticity and two articles (39, 43) included patients with upper limb spasticity. The fundamental features of the included research were outlined in Table 1. With regard to the outcome measure, all included articles reported the MAS. The characteristics of participants, vibration parameters, and intervention schemes are outlined in Table 2.

**Table 1:** General characteristics of the studies.

**Included study**	**Year**	***n* (experiment)**	***n* (control)**	**Age/mean (SD)**	**Spasticity sites**
Liao et al. (41)	2016	56	28	61.2 (9.2)/59.8 (9.1)	Lower limb
Lee et al. (39)	2016	15	15	59.2 (7.72)/60.24 (6.73)	Upper limb
Pang et al. (13)	2013	41	41	57.3/57.4	Lower limb
Alp et al. (23)	2018	10	11	61.2 (11.043)/62.91 (8.154)	Lower limb
Brogårdh et al. (26)	2012	16	15	61.3 (8.5)/63 (5.8)	Lower limb
Hwang (42)	2018	9	9	66 (5.77)/70.25 (2.07)	Lower limb
Wang et al. (43)	2018	17	16	48.35 (6.87)/49.17 (7.33)	Upper limb
Li et al. (40)	2014	23	22	49.23 (11.31)/47.43 (11.39)	Lower limb
Wei (44)	2019	20	21	57.25 (7.97)/58.1 (8.49)	Lower limb
He (38)	2020	20	20	54.25 (9.22)/59.9 (7.62)	Lower limb
Xiao et al. (37)	2022	25	25	63.53 (5.26)/63.62 (4.21)	Lower limb

**Table 2:** Participant characteristics, vibration parameters, and interventions.

**Included study**	**Participant characteristics**	**Vibration parameters**	**Interventions**	**Outcome**	**Measurement time points**	**Adverse events**	**Follow-up time**
Liao et al. (41)	Gender(M/F): 62/22	Frequency: 20/30 Hz	VG1: HWBV + Dynamic exercise 15 min × 3/weeks × 30	MAS, Isokinetic testing	Within 1 week after 30 treatment sessions	One participant from the LWBV group reported mild knee pain after WBV therapy and five reported fatigues	/
	Mean age: 61.2	Amplitude: 1 mm	VG2: LWBV + Dynamic exercise 15 min × 3/weeks × 30				
	Time since stroke: >6 months	Duration of the vibration: 15 min	CG: Dynamic exercise 15 min × 3/weeks × 30				
			Position: stand position				
Lee et al. (39)	Gender (M/F): 24/21	Frequency: 5–15 Hz	VG1: WBV + TRT 60 min × 3/weeks × 4 w	MAS, FMA, Maximal grip strength	4 weeks	/	/
	Mean age: 59.3	Amplitude: 1–6 mm	VG2: WBV + TUE 60 min × 3/weeks × 4 w				
	Time since stroke: >6 months	Duration of the vibration: 30 min	CG: TUE 60 min × 3/weeks × 4 w				
			Position: seated in front of the platform				
Pang et al. (13)	Gender (M/F): 58/24	Frequency: 20–30 Hz	VG: WBV + six different exercises 15 min × 3/weeks × 8 w	MAS, Isokinetic testing	8 weeks	Not reported	3 months
	Mean age: 57.35	Amplitude: 0.44–0.6 mm	CG: Sham vibration + six different exercises 15 min × 3/weeks × 8 w				
	Time since stroke: >6 months	Duration of the vibration: 15 min	Position: stand position				
Alp et al. (23)	Gender (M/F): 19/2	Frequency: 40 Hz	VG: WBV + exercise 20 min × 3/weeks × 4 w	MAS, FIM, 10 mWT	4 weeks	/	3, 6 months
	Mean age: 60.1	Amplitude: 4 mm	CG: Sham vibration + exercise 20 min × 3/weeks × 4 w				
	Time since stroke: >12 months	Duration of the vibration: 5 min	Position: stand position				
Brogårdh et al. (26)	Gender (M/F): 25/6	Frequency: 25 Hz	VG: WBV 2/weeks × 6 w	MAS, BBS, Gait performance, stroke impact scale	6 weeks	Not reported	/
	Mean age: 62.15	Amplitude: 3.75 mm	CG: Sham vibration 2/weeks × 6 w				
	Time since stroke: >6 months	Duration of the vibration:7 min	Position: stand position				
Hwang (42)	Gender (M/F): 10/8	Frequency: 20–30 Hz	VG: WBV + CPT 30 min × 5/weeks × 4 w	MAS, BBS, MMT, FAC, MBI	4 weeks	Not reported	
	Mean age: 68.1	Amplitude: 2–3 mm	CG: CPT 30 min × 5/weeks × 4 w				
	Time since stroke: < 6 weeks	Duration of the vibration: 10 min	Position: stand position				
Wang et al. (43)	Gender (M/F): 27/6	Frequency: 4–6 Hz	VG: WBV + CPT 6/weeks × 4 w	MAS, RMS, FMA, MBI	4 weeks	/	/
	Mean age: 48.8	Amplitude: 4 mm	CG: CPT 6/weeks × 4 w				
	Time since stroke: ≤ 1 month	Duration of the vibration: 10 min	Position: sit on the vibration platform				
Li et al. (40)	Gender (M/F): 34/11	Frequency: 30 Hz	VG: WBV + CPT 6/weeks × 8 w	MAS, FMA, Gait analysis	8 weeks	Not reported	/
	Mean age: 48.3	Amplitude: 0.5 mm	CG: CPT 6/weeks × 8 w				
	Time since stroke: ≤ 1 month	Duration of the vibration: 10 min	Position: stand position				
Wei (44)	Gender (M/F): 28/13	Frequency: 18–25 Hz	VG: WBV + Bobath 55 min × 5/weeks × 4 w	MAS	4 weeks	/	/
	Mean age: 58.1	Amplitude: 9 mm	CG: Bobath 55 min × 5/weeks × 4 w				
		Duration of the vibration: 10 min	Position: stand position				
He (38)	Gender (M/F): 43/17	Frequency: 4 Hz	VG1: WBV + AT + CPT 5/weeks × 4 w	MAS	4 weeks	Not reported	/
	Mean age: 58.1	Amplitude: 4 mm	VG2: WBV + CPT 5/weeks × 4 w				
	Time since stroke: 2 weeks−6 months	Duration of the vibration: 15 min	CG: CPT 5/weeks × 4 w				
			Position: stand position				
Xiao et al. (37)	Gender (M/F): 27/23	Frequency: 20–30 Hz	VG: WBV + ESWT + CPT 5/weeks × 4 w	MAS, BBS, FMA, Gait analysis	4 weeks	/	/
	Mean age: 63.6	Amplitude: 2–3 mm	CG: ESWT + CPT 5/weeks × 4 w				
	Time since stroke: >3 months	Duration of the vibration: 15 min	Position: stand position				

VG, vibration group; CG, control group; TRT, task-related training; TUT, traditional upper training; CPT, conventional physical training; AT: acupuncture therapy; HWBV, high-intensity whole-body vibration; LWBV, low-intensity whole-body vibration; MAS, Modified Ashworth Scale; UDRS, Unified trunk assessment for dystonia; FMA, Fugl-Meyer exercise score; 10 mWT, 10-m walking test; FIM, Functional Independence Scale; RMS, root mean square value of surface EMG; MBI, modified Barthel index; PASS, score for posture control; ESWT, extracorporeal shock wave therapy; BBS, Berg Balance Scale; FAC: functional ambulation category scale.

### 3.3. Risks of bias and level of evidence

**Figure 2:**
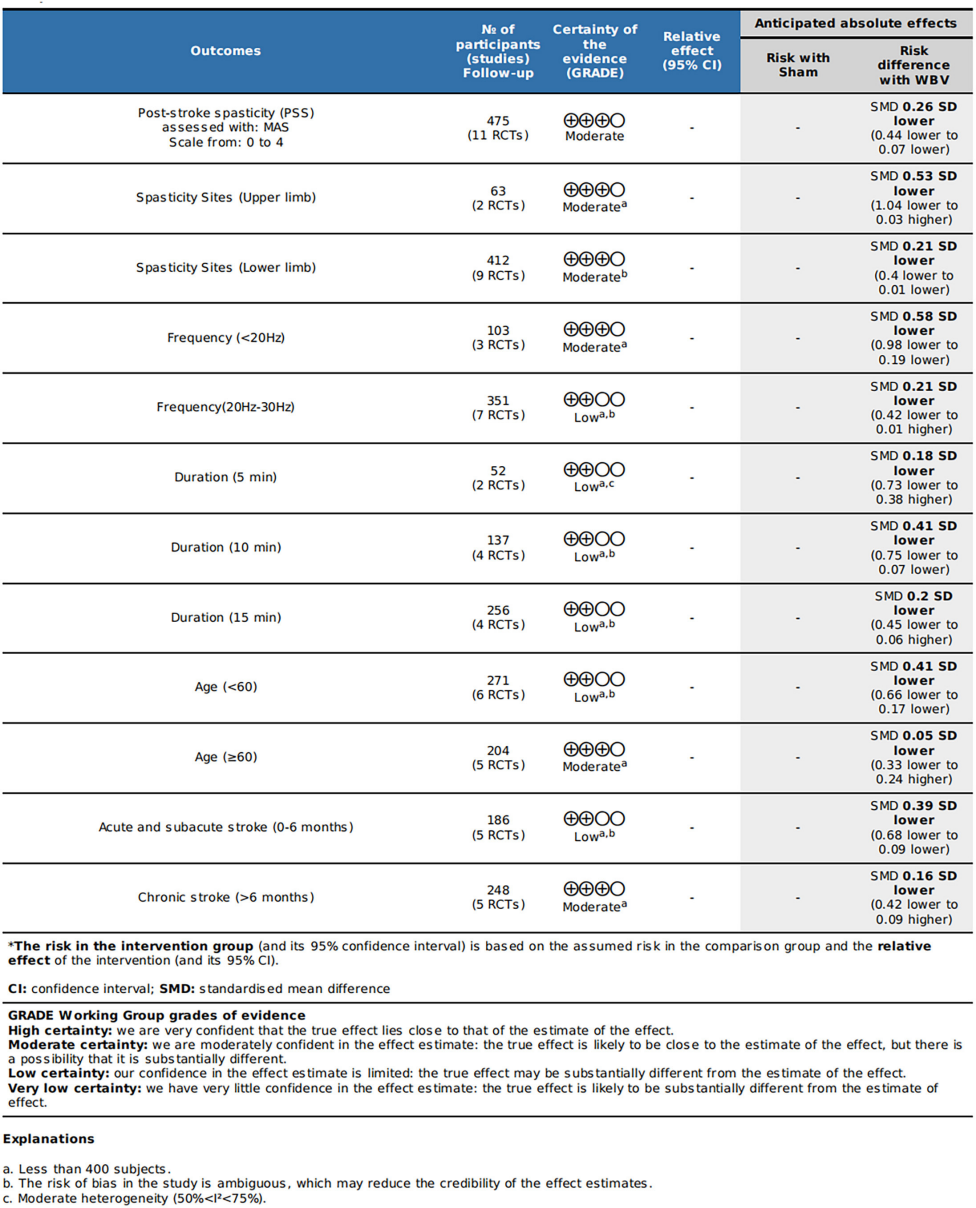
Quality of evidence for the included studies (GRADE).

We discovered moderate-quality evidence for the possible impact of WBV in alleviating spasticity based on the evaluation criteria. We discovered that the reliability of the evidence was negatively impacted by the possibility of uncertainty bias and a limited number of included participants. As shown in Figure 2, there was moderate-quality evidence that WBV might have an impact on the decrease of spasticity. The evidence's quality was diminished by the uncertain danger of bias and the imprecision of the participants included in subgroups.

**Figure 3:**
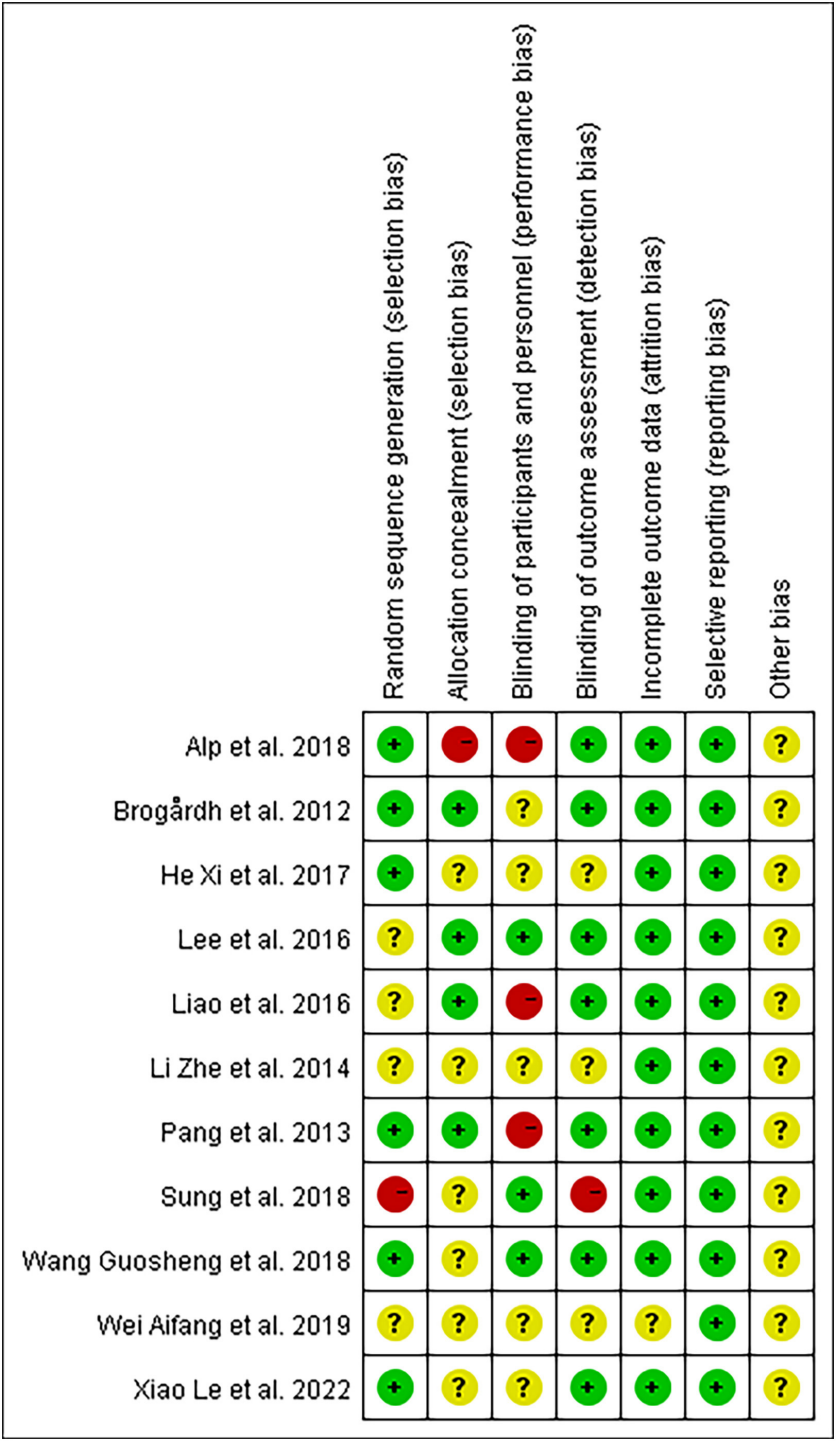
Summary of bias risk.

**Figure 4:**
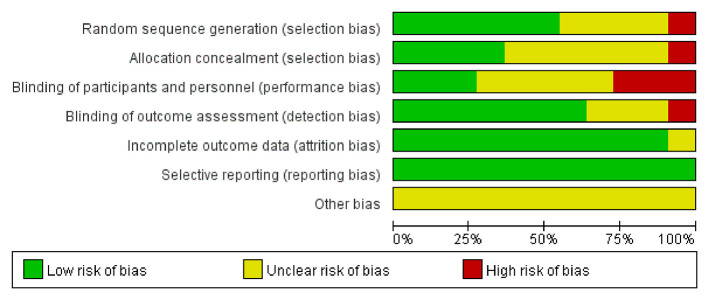
Bias risk bar chart.

The evaluation of the potential for bias in the included studies is summarized in Supplementary Table S1. Overall, according to the Cochrane Bias Risk Scale, four studies (13, 26, 39, 43) met five low-bias risk criteria, three studies (23, 37, 41) met four criteria, two studies (38, 42) met three criteria, one study (40) met two criteria, and one study (44) only met one low-bias standard. We summarized the results as follows: (1) Random sequence generation: except Sung (42) using a randomization method of high bias risk, all of the other included studies described a method of random sequence generation; (2) Allocation hidden: four studies (13, 26, 39, 41) reported a clear random assignment scheme and the rest of studies did not report; (3) Implementation bias and measurement bias: eight studies and four studies, respectively, were found to have a low risk of implementation and measurement bias. The studies by Wei (44), Li et al. (40), and He (38) were unclear in these two respects: (4) Follow-up bias: only the studies by Wei (44) indicated missing data, which means the remaining were deemed to low risk; (5) Reporting bias and other bias: other possible sources of bias could not be found, and not all research used selective reporting (Figures 3, 4).

### 3.4. Meta-analysis

#### 3.4.1. Overall meta-analysis

**Figure 5:**
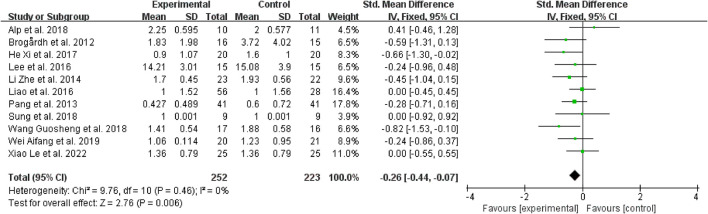
Forest plot analysis of the efficacy of WBV on spasticity compared with control.

To explore the effectiveness of WBV in relieving spasticity, it was known from the forest plot analysis that when compared to the control group, WBV or WBV used in conjunction with other intervention modalities was linked to a reduction in muscular spasticity (SMD = −0.26, 95% CI: −0.44 to −0.07, *P* = 0.006) (Figure 5). An included research (37) comparing the effect of WBV combined with extracorporeal shock wave therapy on spasticity and sham stimulation combined with extracorporeal shock wave therapy. According to this study, when compared to other therapies, the use of WBV together with extracorporeal shock wave therapy significantly improves the curative outcomes.

#### 3.4.2. Subgroup analysis based on the course of stroke

**Figure 6:**
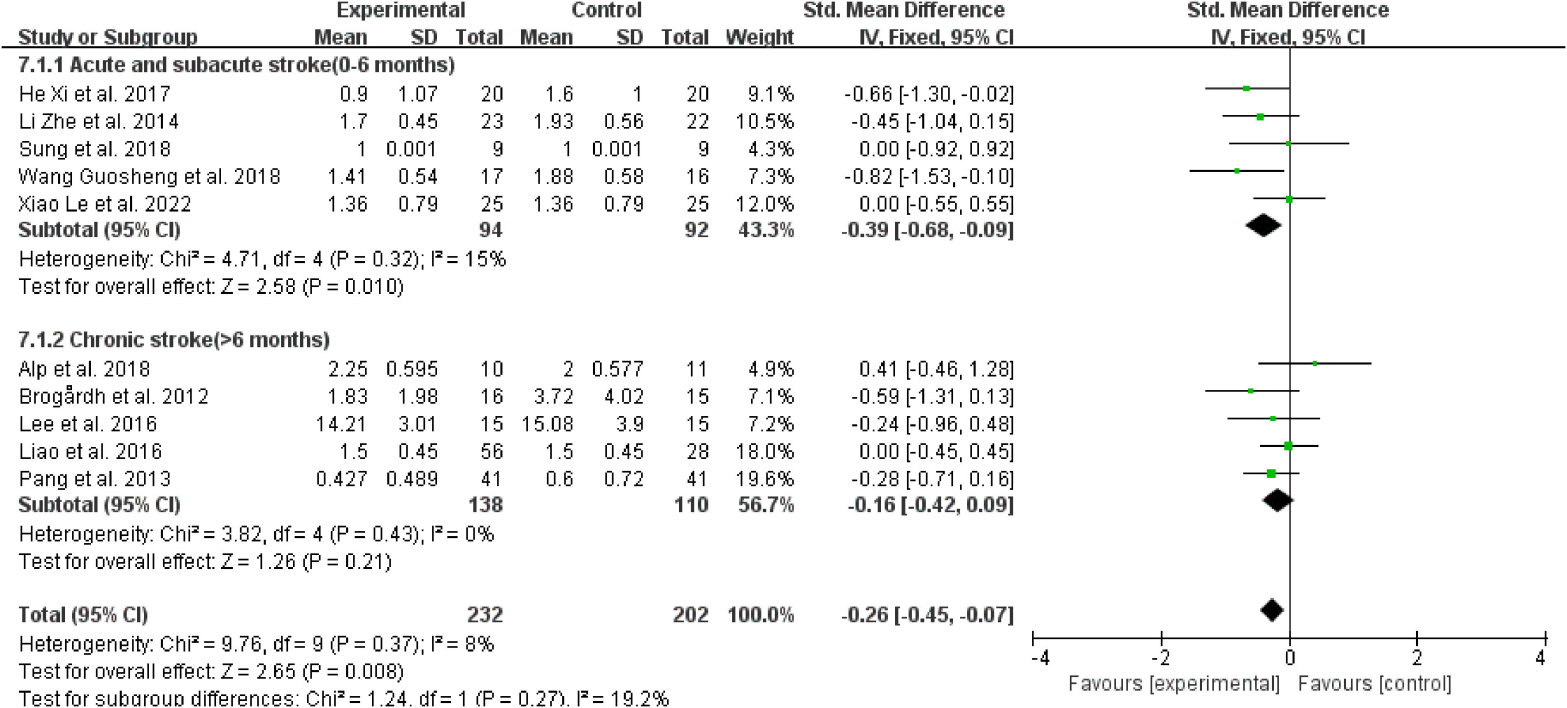
Forest plot analysis of the efficacy of WBV in subgroups divided on the course of a stroke.

Regarding the course of a stroke, WBV did not significantly reduce spasticity in patients with chronic stroke (>6 months) vs. the control group (SMD = −0.16, 95% CI: −0.42 to 0.09, *P* = 0.21) (Figure 6); however, for those with acute and subacute stroke (0–6 months), it dramatically improved the outcomes (SMD = −0.39, 95% CI: −0.68 to −0.09, *P* = 0.01) (Figure 6).

#### 3.4.3. Subgroup analysis based on the age

**Figure 7:**
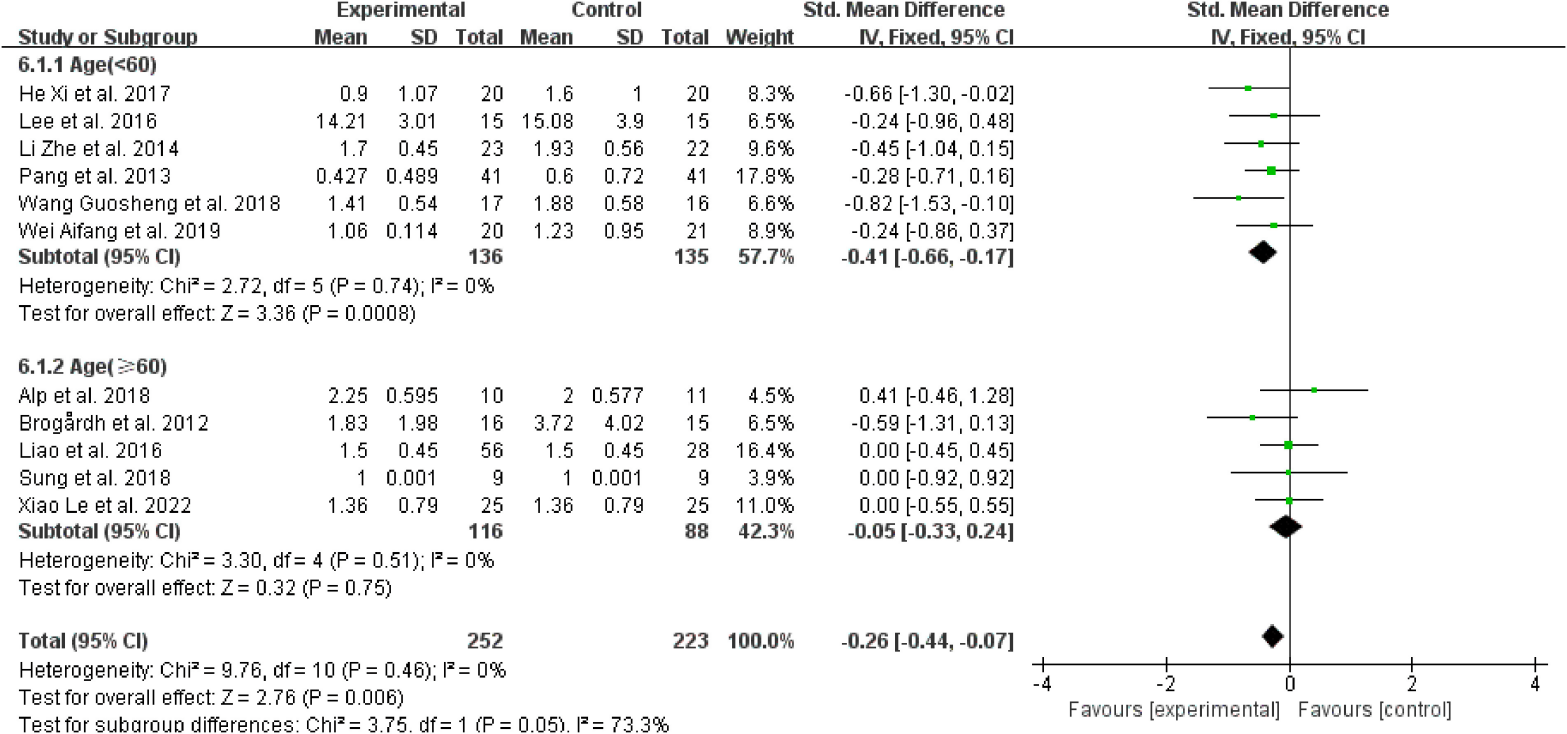
Forest plot analysis of the efficacy of WBV in subgroups divided on the basis of age.

Regarding age, for patients younger than 60 years, spasticity was dramatically reduced in contrast to the control group when WBV was added to other therapies (SMD = −0.41, 95% CI: −0.66 to −0.17, *P* = 0.0008) (Figure 7), while those older than 60 did not have the same improvement (SMD = 0.05, 95% CI: −0.33 to 0.24, *P* = 0.75) (Figure 7).

#### 3.4.4. Subgroup analysis based on spasticity sites

**Figure 8:**
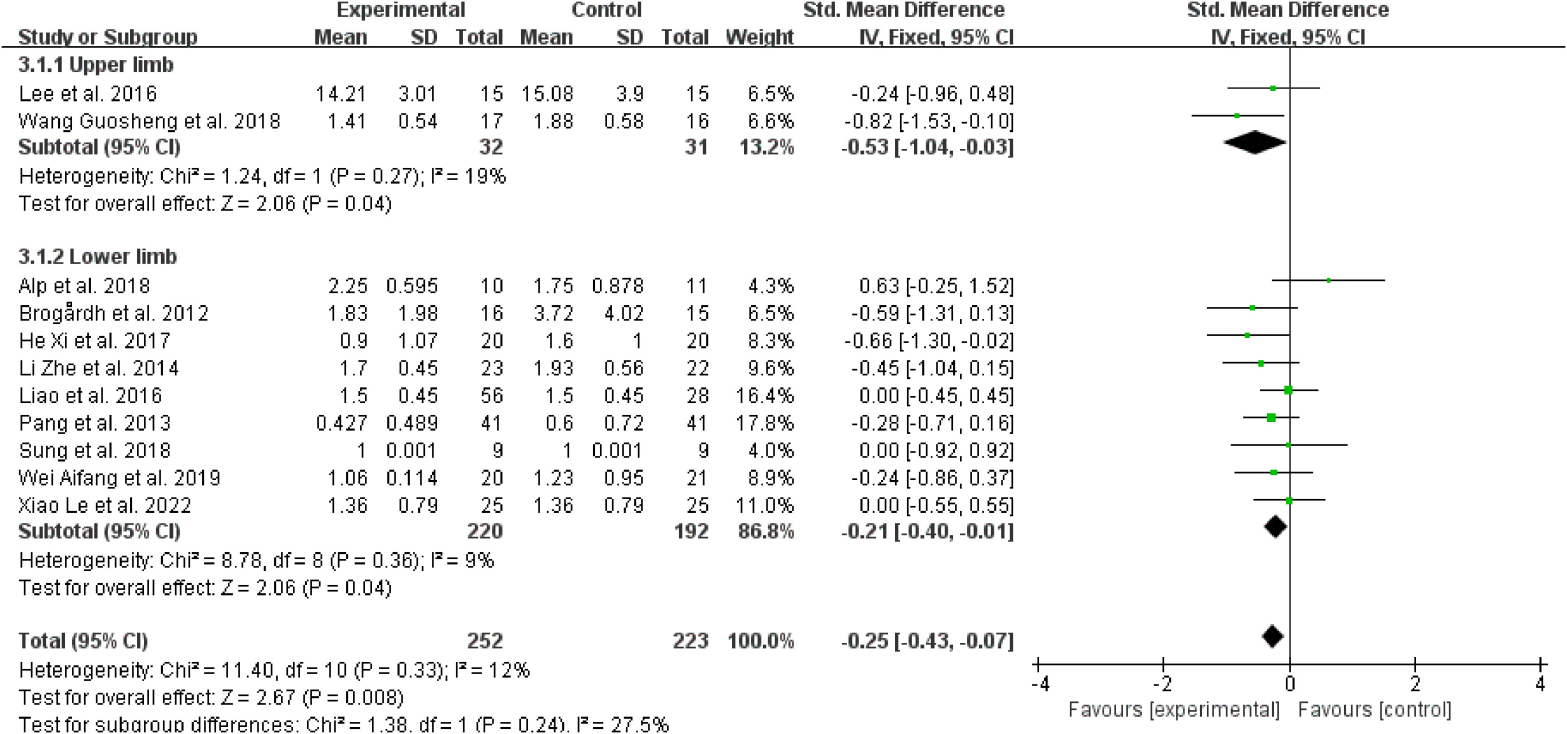
Forest plot analysis of the efficacy of WBV in subgroups divided on the basis of spasticity sites.

Regarding spasticity sites, for patients with upper limb spasticity, WBV was more effective than the control treatments when added to other treatments (SMD = −0.53, 95% CI: −1.04 to 0.03, *P* = 0.03) (Figure 8); for patients with a lower limb (SMD = −0.21, 95% CI: −0.40 to −0.01, *P* = 0.04), WBV was added to other therapies, and it also outperformed the control treatments (Figure 8).

#### 3.4.5. Subgroup analysis based on vibration frequency

**Figure 9:**
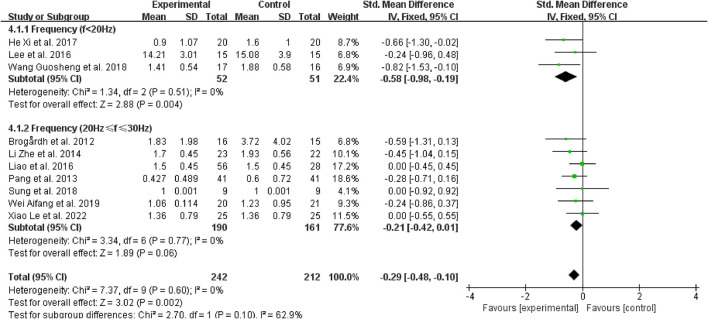
Forest plot analysis of the efficacy of WBV in subgroups divided on the basis of vibration frequency.

Regarding the vibration frequency selected in the included study, it was also found that the signal distortion of the high-frequency vibration (>30 Hz) was more serious, while WBV frequencies below 20 Hz may result in resonance effects, amplifying the vibration signal and perhaps having negative effects (45). Therefore, we chose to bounded 20 and 30 Hz. Three studies revealed that vibration frequencies below 20 Hz were superior for reducing post-stroke spasticity (38, 39, 43) (SMD = −0.58, 95% CI: −0.98 to −0.19, *P* = 0.004) (Figure 9). However, for frequencies between 20 Hz and 30 Hz (SMD = −0.21, 95% CI: −0.42 to 0.01, *P* = 0.06), other therapies did not benefit from the inclusion of WBV over the control treatments (Figure 9).

#### 3.4.6. Subgroup analysis based on vibration duration

**Figure 10:**
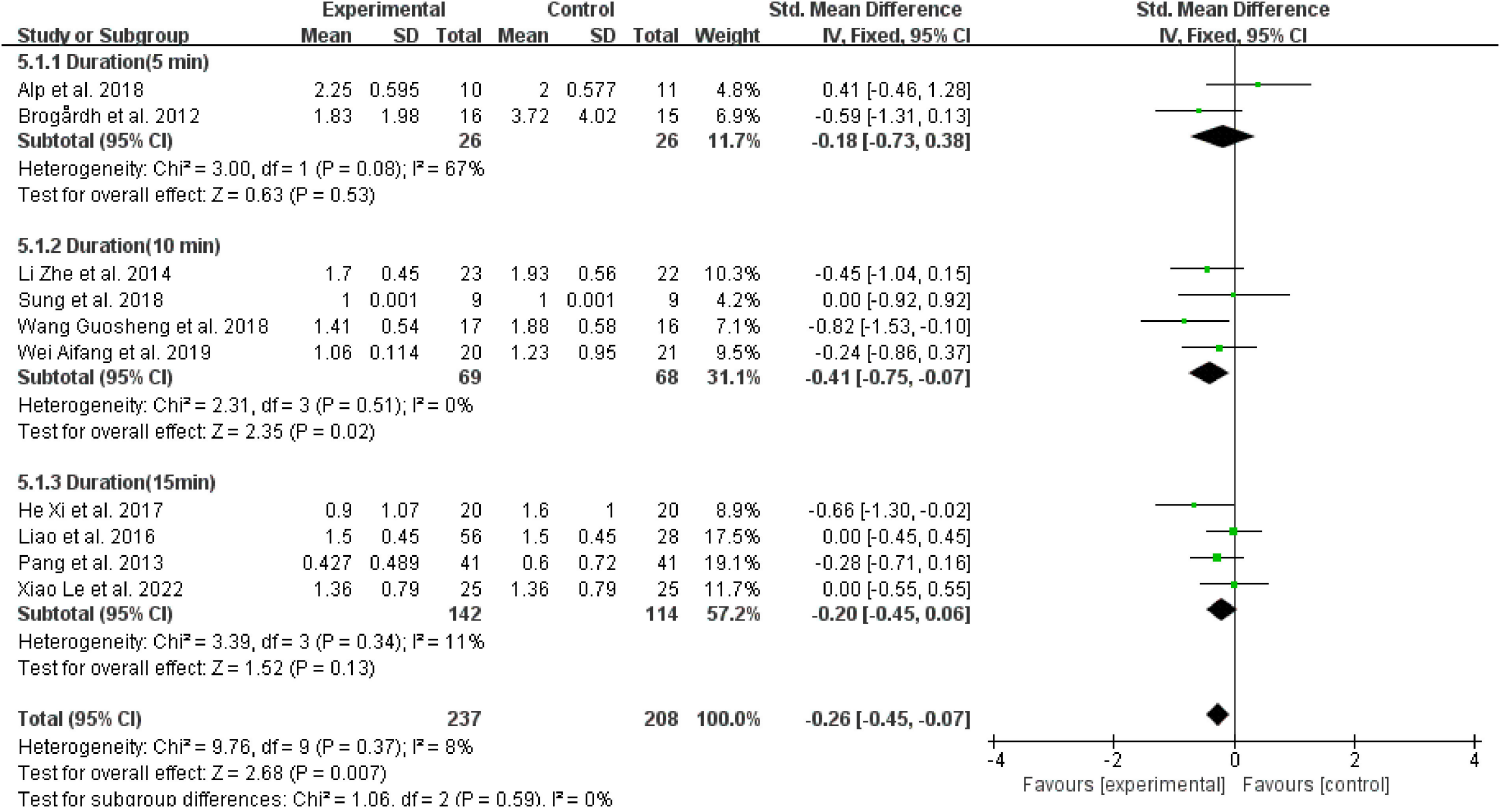
Forest plot analysis of the efficacy of WBV in subgroups divided on the basis of vibration duration.

Regarding vibration duration, four studies (40, 42–44) performed 10 min of vibration, and the effects of WBV were better than those applied 5 and 15 min of vibration to patients with spasticity (SMD = −0.41, 95% CI: −0.75 to −0.07, *P* = 0.02) (Figure 10).

### 3.5. Long-term effects and adverse effects

A follow-up evaluation of WBV for spasticity post-stroke was only reported in two randomized clinical trials (13, 23). Pang et al. (13) reported that knee spasticity levels had a decreasing trend and the MAS score was significantly lower than baseline at 1 month after WBV, the MAS score of the ankle joint, however, did not significantly change over time. Alp et al. (23) reported that ankle spasticity levels decreased gradually at the 3- and 6-month follow-ups. As for the adverse effects, in five studies, no notable serious adverse events associated with WBV were reported (13, 26, 38, 40, 42). According to one study (41), five modest side effects (fatigue, redness of the skin, mild headache, and drowsiness) and 1 out of 84 patients experienced mild knee pain following the WBV.

### 3.6. Publication bias

For each observation index, funnel plots were produced (Supplementary Figure S2). The funnel plots show that the included studies were generally symmetric and focused, indicating that there was little to no indication of publication bias.

## 4. Discussion

This meta-analysis was intended to assess the effectiveness and safety of WBV in treating individuals with post-stroke spasticity, and simultaneously, an appropriate application scheme was also explored by analyzing some possible influencing parameters or factors. Overall, the analysis comprised a total of 11 trials with 475 individuals. We identified moderate-quality proof that WBV was regarded as a safe and effective adjunctive therapy in patients with post-stroke spasticity, especially when used at a vibration frequency below 20 Hz for 10 min, for patients with a stroke under the age of 60 years who have post-stroke spasticity in their upper and lower limbs.

As mentioned in a prior review (24), it came to the conclusion that there was weak proof that short-term WBV therapy lowers lower limb spasticity in patients suffering neurological disorders, which was consistent with our conclusion. Lucrezia et al. (46) also found in contrast to chronic patients, those who were acute or subacute appear to benefit from vibration therapy more. However, the appropriate WBV treatment parameters for individuals with post-stroke spasticity have not been identified by prior investigations. Compared with early reviews (24, 46), this review conducted a more comprehensive subgroup analysis of the factors that may affect treatment efficacy, including age, stroke course, and vibration parameters (frequency, time, and location). Given the available research data, our study offered safe and effective WBV settings to relieve post-stroke spasticity despite the lack of high-quality evidence. However, a different systematic review (47) found inadequate data to either support or disprove the claim that WBV can relieve spasticity in patients with stroke. The short number of studies included the low number of studies that produced statistically significant outcomes, and the wide range of intervention strategies may be responsible for this conclusion. As the studies evaluating how WBV affects muscular spasticity post-stroke have significantly increased, we believe that the conclusions of this study need to be further updated with increasing evidence on the effectiveness of WBV for spasticity today.

As far as we are aware, this is the first meta-analysis to thoroughly compile and evaluate the effectiveness and safety of WBV in the treatment of post-stroke spasticity; besides, some influencing parameters and factors were also explored, offering support for the clinical application of WBV. Furthermore, from the results of the subgroup analysis, we filled the current gap in the clinical use of WBV in post-stroke spasticity treatment by offering a reliable and secure prescription for it. The Cochrane Collaboration's guidelines and criteria were strictly adhered to in this meta-analysis (31). In addition, as determined by the strict inclusion and exclusion standards, the most relevant randomized clinical studies were included. To prevent conclusions from being biased or misleading, we evaluated the quality of the evidence using GRADEpro GDT (48). Subgroup analysis of the moderate quality of evidence revealed WBV can reduce spasticity in the upper and lower limb after stroke, which was consistent with the conclusion of previous systematic reviews (49, 50). However, current studies have focused on the upper and lower limbs, and there are few studies on WBV for trunk muscle spasticity, and the effectiveness of trunk spasms is difficult to conclude at present. Therefore, to better comprehend how WBV affects post-stroke spasticity in other clinically relevant body parts, especially the trunk, additional well-designed randomized clinical trials are required.

This review found low-quality evidence that patients with both acute and subacute stroke can benefit from WBV for post-stroke spasticity. Considering that spasticity gradually increased within 1 month of onset, while stroke survivors often present with limb weakness within 3 months (5), in survivors with acute and subacute stroke, the spasticity reduction would have been more considerable. According to certain studies (50, 51), patients with an acute stroke who receive low vibration frequency (20 Hz) had stronger muscles than those in the control group. However, this conclusion required validation with more and higher quality RCTs due to the low quality of the literature evidence. Due to the low quality of the literature data, this finding needed to be validated with more randomized controlled trials of higher quality. Moreover, varying degrees of impairment may have different effects on soft tissue's properties, such as how much muscle atrophy, how much muscle turns into connective tissue, and how much sarcomere was lost (3), thus limiting the application of WBV.

The present study found moderate-quality evidence that WBV could effectively improve post-stroke spasticity at frequencies below 20 Hz. Some studies (41, 52) suggested that an enhanced vibration signal and possible negative effects can emerge from vibrations at frequencies below 20 Hz because of a significant resonance effect, such as internal organ damage. However, three of the included studies (37, 39, 44) used vibrations below 20 Hz, among which only a few produced mild adverse effects, and spasticity alleviation outperformed that of the control group by a large margin. Considering the outcomes of this study, WBV at frequencies < 20 Hz can be applied to ameliorate post-stroke spasticity. We did not decide to carry out a subgroup analysis for the study with a frequency higher than 30 Hz because of the small number of inclusions. Since their greater peak acceleration values, high-frequency vibrations (>30 Hz) have the potential to cause damage (45), which may not be appropriate for patients with chronic stroke, many of whom have frail bones (53). Furthermore, the only included study (41) reported that some patients showed fatigue and discomfort at high frequency vibration. Some studies (54, 55) have shown that spasticity can be effectively treated by inducing frequencies between 20 and 30 Hz. Therefore, to strengthen the evidence for therapeutic application, more study is needed to confirm the impact and mechanism of different vibration frequencies on post-stroke spasticity.

Regarding the WBV duration, post-stroke spasticity can be effectively alleviated for the last 10 min. Four included trials (40, 42–44) that adopted a 10-min vibration time reported significant effects on spasticity improvement, and no withdrawal from the trial due to adverse effects. Hence, the present review suggests that 10 min of WBV as an adjunctive therapy was safe and effective for improving post-stroke spasticity.

Another important parameter is the posture of the patient. Due to the limited number of inclusions, only a systematic evaluation of patient posture was performed. Patient postures in 10 studies were standing posture, nine of which measured lower limb spasticity, and one measured trunk spasticity. In the other two studies, patients were in a seated posture, which studied upper limb spasticity. In research involving WBV training in a standing position, the majority of patients were asked to perform static or dynamic semi-squat training instead of a static upright standing station. Studies have found that static upright stations were prone to transmitting vibration to the head, which can lead to adverse reactions such as dizziness (56). There is plenty of evidence that knee flexion angles may impact the transmission of vibration to the head, which should be avoided. Abercromby et al. (57) demonstrated that when the knee flexion angle rises from 10 to 30, it appears that head transmissibility decreases, using a fixed setup (30 Hz and 4 mm). It can also be utilized with dynamic motions for WBV. The response to dynamic exercise was equivalent to static postures, according to a recent study (58) that looked at transmissibility to the head (frequencies between 20 and 50 Hz) during dynamic squats. Therefore, a certain knee flexion Angle can reduce the transmission of vibration to the head to reduce adverse reactions. Moreover, Boo et al. (59) found that chronic stroke patients with whole-body vibration training in a seated position had increased muscle tone and upper limb function. This may be related to an improvement in the postural control ability. Improving postural control can make arm movement more flexible and improve activities of daily living (60). Verheyden et al. (61) reported that sitting training was effective in improving postural control. Hence, to enhance upper limb function, it was advised to carry out rehabilitation activities while seated on shaky or moving surfaces, such as the vibration platform.

There are certain restrictions on the current meta-analysis. First, all the participants of this study were patients with mild to moderate spastic stroke. There has not been enough research done on the effectiveness of WBV in patients suffering from severe spasticity, thus, this conclusion may not be applied and extended to all patients with post-stroke spasticity. Second, the potential long-term effects of WBV cannot be determined because only two studies had a 3–6-month followed-up. Third, due to a variety of mixed factors, the results can be biased to some extent. These limitations, to some extent, limit the applicability of WBV and the credibility of the conclusions, and the conclusions drawn should be treated with caution. To assess how different vibrational parameters affect post-stroke spasticity, create optional treatment protocols, and provide scientific justification for the purpose of WBV therapeutic usage in the management of post-stroke spasticity, additional prospective studies are required.

## 5. Conclusion

Based on current moderate evidence, it seems when used as an adjuvant therapy for 10 min at a frequency of < 20 Hz, whole-body vibration has been proven to work best for upper and lower limb spasticity in patients with acute and subacute strokes under the age of 60 years. Studies of better quality are required in the future to examine its long-term safety and effectiveness, as well as the mechanism of action.

## Data availability statement

The original contributions presented in the study are included in the article/Supplementary material, further inquiries can be directed to the corresponding authors.

## Author contributions

QZe, JZ, and XZ: conception and design and typographical logic of the article. YZ, LC, and GL: literature selection and acquisition of data. QZh, SZ, and ShuL: analysis and interpretation of data and editing the article. ShiL, LH, and SC: study supervision and revising the article. All authors contributed to the article and approved the submitted version.
