# Supplementary material for: Efficacy and safety of whole-body vibration therapy for post-stroke spasticity: A systematic review and meta-analysis
Source: Front Neurol. 2023 Jan 26;14:1074922. doi: 10.3389/fneur.2023.1074922 (PMC9909105; doi:10.3389/fneur.2023.1074922)
Supplement: Supplementary file 1 [file Table_1.docx]

**Supplementary Table S1.** Cochrane Bias Risk Scale scores of included studies

| **Included study** | **Random sequence generation** | **Distribution hidden** | **Implementation of bias** | **Measurement bias** | **Follow-up of bias** | **Reporting bias** | **Other bias** |
| --- | --- | --- | --- | --- | --- | --- | --- |
| Liao 2016 (41) | **?** | **+** | **-** | **+** | **+** | **+** | **?** |
| Lee 2016 (39) | **?** | **+** | **+** | **+** | **+** | **+** | **?** |
| Pang 2013 (13) | **+** | **+** | **-** | **+** | **+** | **+** | **?** |
| Alp 2018 (23) | **+** | **-** | **-** | **+** | **+** | **+** | **?** |
| Brogårdh 2012 (26) | **+** | **+** | **+** | **+** | **+** | **+** | **?** |
| Sung 2018 (42) | **-** | **?** | **+** | **-** | **+** | **+** | **?** |
| Wang Guo-sheng 2018 (43) | **+** | **?** | **+** | **+** | **+** | **+** | **?** |
| Li Zhe 2014 (40) | **?** | **?** | **?** | **?** | **+** | **+** | **?** |
| Wei Aifang 2019 (44) | **?** | **?** | **?** | **?** | **?** | **+** | **?** |
| He Xi 2020 (38) | **+** | **?** | **?** | **?** | **+** | **+** | **?** |
| Xiao Le 2022 (37) | **+** | **?** | **?** | **+** | **+** | **+** | **?** |
| + = low risk; ? = unclear risk; - = high risk | | | | | | | |
