# Supplementary figures and images for: Efficacy and safety of whole-body vibration therapy for post-stroke spasticity: A systematic review and meta-analysis
Source: Front Neurol. 2023 Jan 26;14:1074922. doi: 10.3389/fneur.2023.1074922 (PMC9909105; doi:10.3389/fneur.2023.1074922)

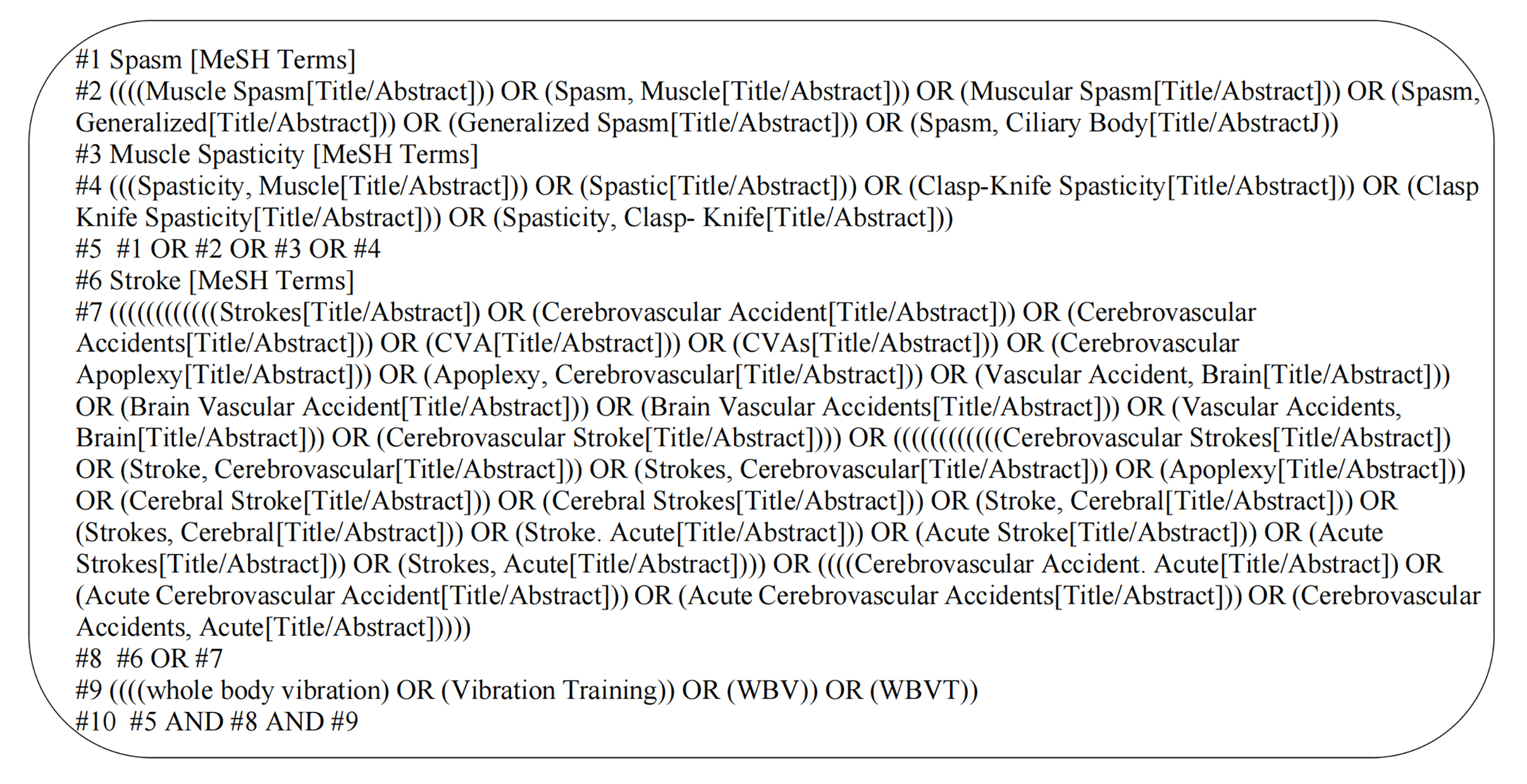

Supplement: Supplementary file 2 [file Image_1.TIF]

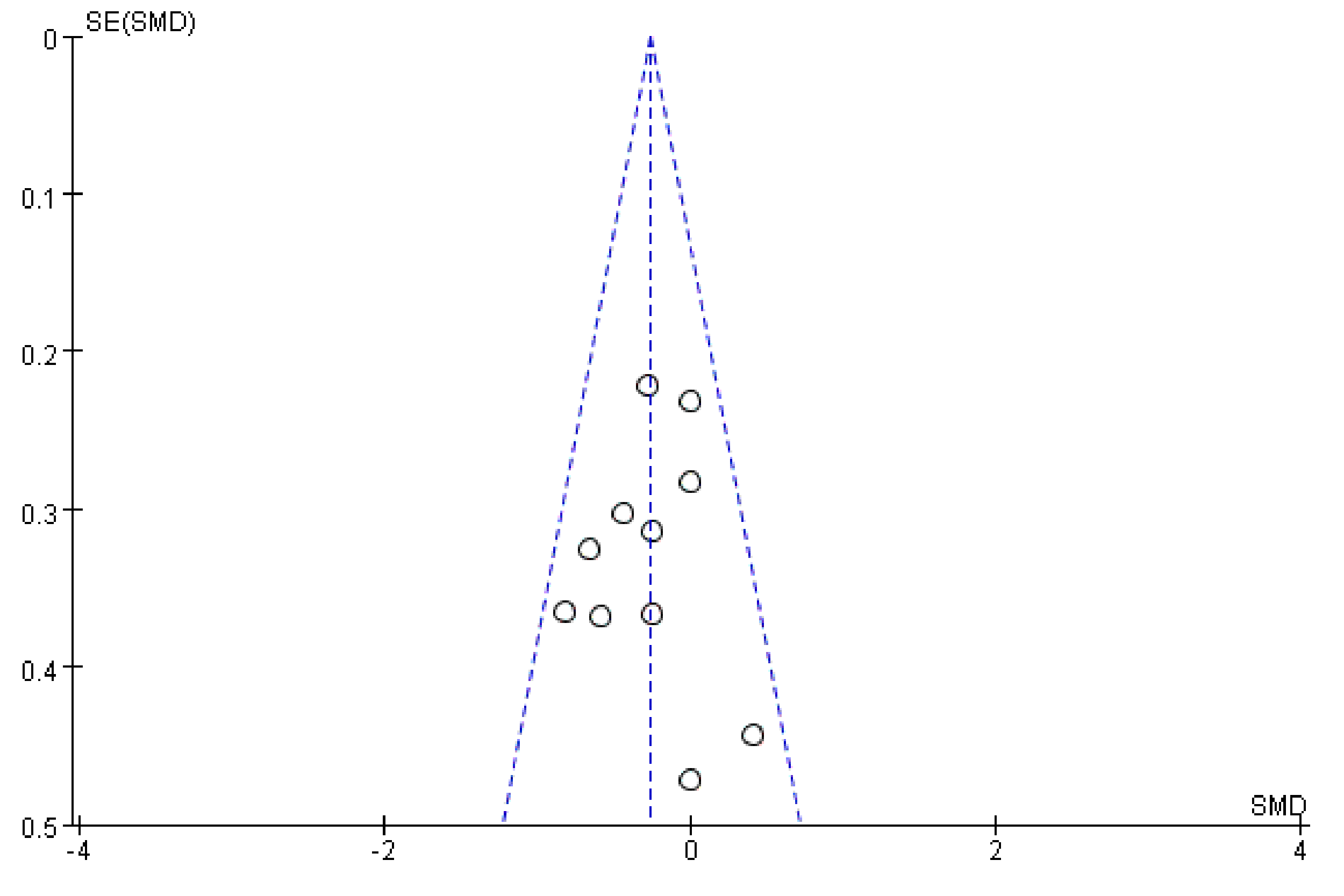

Supplement: Supplementary file 3 [file Image_2.TIF]
